# Comprehensive brain tissue metabolomics and biological network technology to decipher the mechanism of hydrogen-rich water on Radiation-induced cognitive impairment in rats

**DOI:** 10.1186/s12860-023-00491-4

**Published:** 2023-09-26

**Authors:** Xiaoming Liu, Mengya Liu, Huan Liu, Hui Yuan, Yong Wang, Xiaoman Chen, Jianguo Li, Xiujun Qin

**Affiliations:** 1 Department of Radiology and Environmental Medicine, China Institute for Radiation Protection, CAEA Center of Excellence on Nuclear Technology Applications for Non-Clinical Evaluation for Radiopharmaceutical, Shanxi Key Laboratory for Pharmaceutical Toxicology & Radiation Injury Pharmaceuticals, CNNC Key Laboratory for Radiotoxicology and Preclinical Assessment of Radiopharmaceuticals, Taiyuan, 030006 P. R. China; 2 School of forensic medicine, Shanxi Medical University, Taiyuan, 030001 P. R. China

**Keywords:** Hydrogen-rich water, Ionizing radiation, Brain tissue metabolomics, Biological network

## Abstract

**Background:**

Hydrogen-rich water (HRW) has been shown to prevent cognitive impairment caused by ionizing radiation. This study aimed to investigate the pharmacological effects and mechanisms of HRW on ionizing radiation by coupling the brain metabolomics and biological target network methods.

**Methods and results:**

HRW significantly improves the cognitive impairment in rats exposed to ionizing radiation. Based on metabolomics and biological network results, we identified 54 differential metabolites and 93 target genes. The KEGG pathway indicates that glutathione metabolism, ascorbic acid and aldehyde acid metabolism, pentose and glucuronic acid interconversion, and glycerophospholipid metabolism play important roles in ionizing radiation therapy.

**Conclusion:**

Our study has systematically elucidated the molecular mechanism of HRW against ionizing radiation, which can be mediated by modulating targets, pathways and metabolite levels. This provides a new perspective for identifying the underlying pharmacological mechanism of HRW.

## Introduction

Ionizing radiation can produce a variety of free radicals, damage nucleic acids, proteins, carbohydrates and lipid compounds, thereby destroying the structure and function of cells [1, 2]. Approximately 60–70% of the damage is caused by hydroxyl radicals [3]. The brain contains high levels of polyunsaturated fatty acids (PUFAs) and relatively low levels of antioxidant enzymes, making it highly susceptible to oxidative damage [2]. Radiation can affect cognitive functions such as learning and memory because it can damage neurogenesis, promote brain cell apoptosis, and cause neurodegenerative changes [3]. Radiation may cause long-term cognitive impairment, especially in children whose nervous system is still developing [3, 4].

Hydrogen has great potential in the clinical treatment of various neurological diseases [5]. Previous studies have shown that hydrogen treatment can effectively prevent and alleviate neonatal brain injury and cognitive dysfunction, especially cerebral ischemia-reperfusion injury [6–8]. Hydrogen can cross the blood-brain barrier and quickly cross cell membranes to interact with toxic ROS. Therefore, hydrogen has great potential in the clinical treatment of various diseases of the nervous system.

Metabolomics, as a new omics technique, study the cellular processes involving metabolites, intermediates and products of cell metabolism, and has find applications in drug metabolism, biomarkers, and environmental toxicants. Metabolomics elucidates the end points of biological processes, reflecting the physiological and pathological state through metabolic levels [9]. Therefore, metabolomics is a useful tool to investigate pharmacodynamics and mechanism of action of radioprotectants which reflect the end state of diseases and treatments [10–14]. However, the changes in endogenous metabolites are unknown, and using metabolomics alone as a tool may not reveal the effectiveness of radiation protection agents in the treatment of radiation-induced diseases.

Computer virtual computing based on high throughput omics data analysis and network database search may reveal complex network signal relationships among drugs, targets and diseases [15]. For radioprotective agents, their pharmacological effects are characterized by multi-targets and multi-levels. The biological target network has the advantage to analyze and explain the action mechanisms from a holistic perspective, promoting the in-depth investigation [16]. Combining the two key technologies of computer simulation and metabionomics, the molecular mechanism of agents’ action on diseases can be explained from the metabolic level and genetic levels. For example, Liu et al. linked the biological targets network with metabolomics and found that amino acid metabolism is a potential target for Xiaoyaosan anti-depressant [17].

To the best of our knowledge, no studies have used metabolomics combined with biological network approaches to study the effect of hydrogen on radiation-induced cognitive impairment. This study used metabolomics combined with a biological target network to investigate the molecular biological mechanism of HRW on radiation-induced cognitive impairment in rats.

## Materials and methods

### Materials and reagents

Portable HRW machine (China Institute For Radiation Protection, Shanxi Zhongfu Nuclear Instruments Co.,Ltd.), Linear accelerator (Elekta Synergyt, Crawley, UK), ultra-high-performance liquid chromatography (Dionex Ultimate 3000 UHPLC, Thermo Scientific, USA) coupled to high-resolution mass spectrometry (Q-Exactive Plus, Thermo Scientific, USA). Water, methanol, acetonitrile and formic acid were purchased from CNW Technology Co., Ltd. (Dü sseldorf, Germany). L-2-chlorophenylalanine was obtained from Shanghai Hengchuang Biotechnology Co., Ltd. (Shanghai, China). All chemicals and solvents are of analytical or HPLC grade.

### Animals and ethic statement

Male Sprague-Dawley rats weighing 140–170 g and, aging 6 weeks were purchased from Beijing Vital River Laboratory Animal Technology Co., Ltd., China. Animals were caged in a humidity and temperature-controlled room, with a 12/12 light-dark cycle. Water and food were provided to animals with free access. The animal study protocols were approved by the Animal Protection and Ethics Committee of the China Institute for Radiation Protection. All laboratory operations were performed in accordance with the Regulations of China Institute for Radiation Protection on the administration of laboratory animals. The study was carried out in compliance with the ARRIVE guidelines.

### Establishment of the ionizing radiation model

Twenty-four rats were randomized into four groups (6 in each group), as follows: HRW group received HRW(hydrogen concentration: 0.8–0.9 ppm) without radiation treatment; Radiotherapy group received 30 Gy whole brain irradiation of purified water; Radiation -HRW group received 30 Gy whole brain irradiation with HRW; Control group received purified water without radiation treatment. Rats were dosed with purified water or HRW gavage at 20ml/kg per day 10 min before irradiation and 30 days after irradiation. Immobilize the animal directly with clamps before preparing the animal for radiotherapy with deionized water using a portable HRW machine. Then, at room temperature, a 6-MeV electron beam was emitted at a rate of 3 Gy/min by a linear accelerator to irradiate the whole brain. On the 36th day after irradiation, rats were sacrificed by intraperitoneal injection of pentobarbital and blood was taken from the abdominal aorta, the brains of the animals were collected.

### Morris Water Maze Test

On the 30th day after irradiation, the rats in each group were subjected to a cognitive test in the form of a position navigation test and a spatial search test. In the site navigation task, rats acclimated themselves with the test environment by swimming for 2 min in the circular pool without a platform the day before the formal test. Rats were then trained for five consecutive days. To start training, rats were placed in the pool facing the pool wall, the swimming route of the rats were tracked, the escape latency (the time from entering the water to finding platform–all limbs on the platform) was measured. Rats were allowed to rest on the platform for a few seconds before returning them to their cages. The escape latency was recorded as 120 s if the rat did not find the platform within 2 min and were guided to the platform. The arithmetic mean of the escape latencies was calculated four times per day. The position navigation test was followed by a spatial probe test in which the platform was withdrawn and the rats were placed at any entry point in the pool on day six. The swimming distance of rats were recorded and analyzed within 1 min. Record the passage time they cross the original platform, the time the rat retained in the original platform quadrant, their swimming distance in the platform quadrant, and the number of times the rat crossed the platform.

### Brain tissue samples collection and preparation

After the brain tissue was collected, 30 mg brain tissue sample were accurately weighed and transferred to a 1.5 mL Eppendorf tube. Two small steel spheres and 20 µL of the internal standard (2-chloro-l-phenylalanine in methanol, 0.3 mg/mL) and 400 µL of methanol/water (4/1, v/v) extraction solvent were added to the sample tube. The samples were stored at -80 ℃ for 2 min, ground at 60 HZ for 2 min, sonicated at ambient temperature (25 ℃ to 28 ℃) for 10 min and stored at -20 ℃ for 30 min Centrifuge the extract at 13,000 rpm, 4 ℃ for 15 min. Dry 300 mL of the supernatant in a brown glass vial in a freeze concentration centrifugal dryer. Then 200 µL mixture of methanol and water (1/4, vol/vol) were added to each sample, the sample was vortexed for 30 s and, placed at 4 ℃ for 2 min. Samples were centrifuged at 13,000 rpm, 4 ℃ for 5 min. The supernatant (150 µL) was collected from each tube using a crystal syringe and was filtered through a 0.22 μm microfilter and transferred to an LC vial. Before LC -MS analysis, the vials were stored at -80 ℃ until. QC samples were prepared through mixing aliquots of all samples into a pooled sample.

### Metabolomics analysis

#### UHPLC-MS parameters

Samples were analyzed by ultra-high-performance liquid chromatography coupled to high-resolution mass spectrometry in both ESI positive and ESI negative ion modes. In both positive and negative ionization modes, an ACQUITY UPLC HSS T3 column (100 mm×2.1 mm, 1.8 μm) were employed. Water and acetonitrile, both containing 0.1% formic acid, were used as mobile phases A and B, respectively. The linear gradient was: 0 min, 5% B; 2 min, 5% B; 4 min, 25%B; 8 min, 50% B; 10 min, 80% B; 14 min, 100% B; 15 min, 100% B; 15.1 min, 5%B and 16 min, 5%B. The column temperature was 45 ℃ and the flow rate was 0.35 mL/min. The injection volume was 2 µL. All samples were kept at 4℃ during analysis.

Data acquisition was performed in full scan mode (m/z ranges from 100 to 1000) combined with MSE mode, consisting of alternating acquisition of 2 independent scans with different collision energies (CE) during the run. The mass spectrometry parameters were as follows: a low-energy scan (CE 4 eV) and a high-energy scan (CE ramp 20-45 eV) to fragment the ions. Argon (99.999%) was used as collision-induced dissociation gas; scan rate was 0.2 s/scan; capillary voltages were − 3 kV (negative mode) and 3.8 kV (positive mode); capillary and heater temperatures were 320℃ and 300℃. The auxiliary gas volume flow was 8 arb, sheath gas volume flow was 35 arb. The stepped normalized collision energy (NCE) was set to 10, 20, and 40 eV respectively. The QCs were injected periodically (every 10 samples) throughout the analysis to provide a data set from which repeatability can be assessed.

#### Data processing

The raw data were exported using Xcalibur workstation and imported into Composite Discoverer 2.0 software to obtain matched and aligned peak data. The parameters are as follows: the scanning range is 100–1000 m/z. Ass deviation is 5 ppm. The retention time is 0.05 min. The signal-to-noise ratio threshold is 1.5. The peak data containing retention time, molecular formula, accurate molecular weight, and peak area information were imported into Excel for peak area normalization. Finally, the peak area normalization data were imported into SIMCA-P 14.0 for partial least squares discriminant analysis (PLS-DA) and orthogonal partial least squares discriminant analysis (OPLS-DA). VIP > 1 in the S-plot and *P* < 0.05 in the independent sample *t*-test were used to screen for the most contributing differential metabolites. Differential metabolites were matched and screened using Metlin, HMDB, Pubchem, KEGG, m/zcloud, and others online databases. Finally, the identified differential metabolites were directed to MetaboAnalyst 5.0 for pathway enrichment analysis.

### Construction of biological network of HRW for ionizing radiation therapy

The GeneCards database (https://www.genecards.org/) and the DisGeNET database (http://www.disgenet.org/) were used to identify potential genes associated with ionizing radiation. Metabolites were introduced into the Cytoscape 3.9.2 plug-in MetScape for metabolic enzyme analysis. Finally, Cytoscape 3.9.2 was used to construct a “gene-metabolite” regulatory network for ionizing radiation therapy.

### Biological function and pathway analysis

To elucidate the gene functions and their role in signal transduction, DAVID (https://david.ncifcrf.gov/) was used to evaluate the KEGG and GO enrichment profiles of HRW against ionizing radiation (*P* < 0.05).

### ROC analysis

ROC curve analysis and calculation of AUC were performed using the Omicstudio tool (https://www.omicstudio.cn/home) to screen for potential therapeutic biomarkers and assesse the efficacy of central metabolites in the HRW treatment of ionizing radiation. These metabolites were combined by logistic regression analysis. *P* < 0.05 with a 95% confidence interval was considered statistically significant.

### Statistical analysis

All data were presented as mean ± standard deviation. SPSS 22.0 and GraphPad Prism 7.0 were used for statistical analysis. The independent samples *t* test and one-way analysis of variance were used for comparison between two groups and multiple groups. *P* < 0.05 was considered statistically significant.

## Results

### Effect of HRW on learning and memory in rats with ionizing radiation

**Fig. 1:**
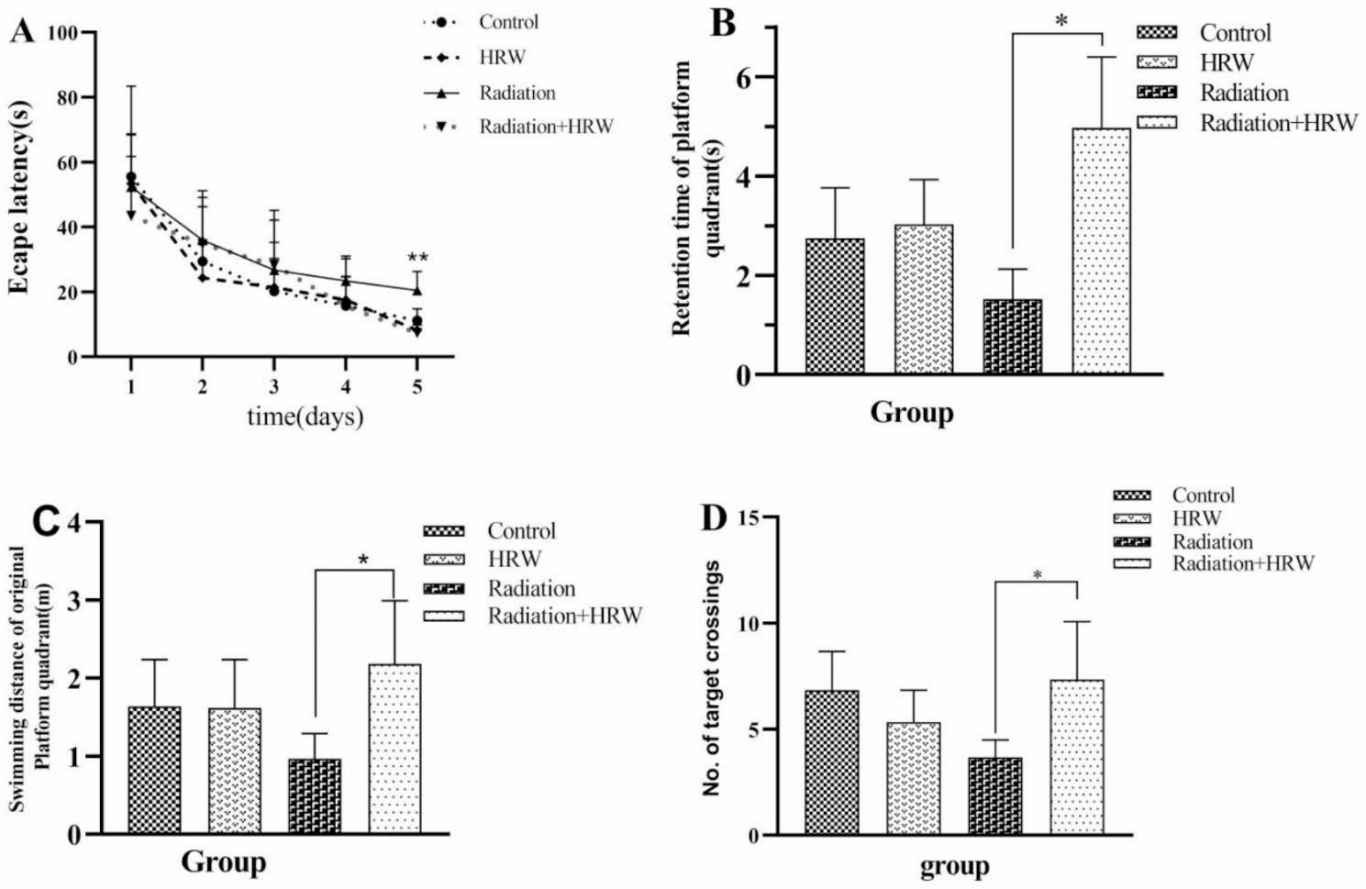
**Effects of hydrogen-rich water (HRW) on cognitive function by Morris water maze test**. (**A**) The escape latencies of different groups during the five-day training period. *P < 0.05 vs. radiation-HRW, one-way ANOVA. (**B**) The retention time in the original platform quadrant. (**C**) The swimming distance in the original platform quadrant. (**D**) The number of platform crossings. *P < 0.05

Morris water maze test was used to assess the functional consequences of radiation exposure on the brain and whether HRW produced any neurocognitive benefits. During the training period of the first five days, the radiation group had significantly longer escape latency than the control group, the HRW group and the radiation -HRW treatment group (*P* < 0.05, Fig. 1A.). The spatial search test was performed from day six. The radiation-HRW group had significantly longer retention time in the original platform quadrant, greater platform crossings, and longer swimming distance than the radiation group (*P* < 0.05 Fig. 1B, C, D). These results indicated that brain radiation damaged the spatial memory and spatial search ability of rats, and HRW had a protective effect on radiation-induced cognitive impairment.

### HRW regulated metabolic disorders in ionizing radiation rats

**Fig. 2:**
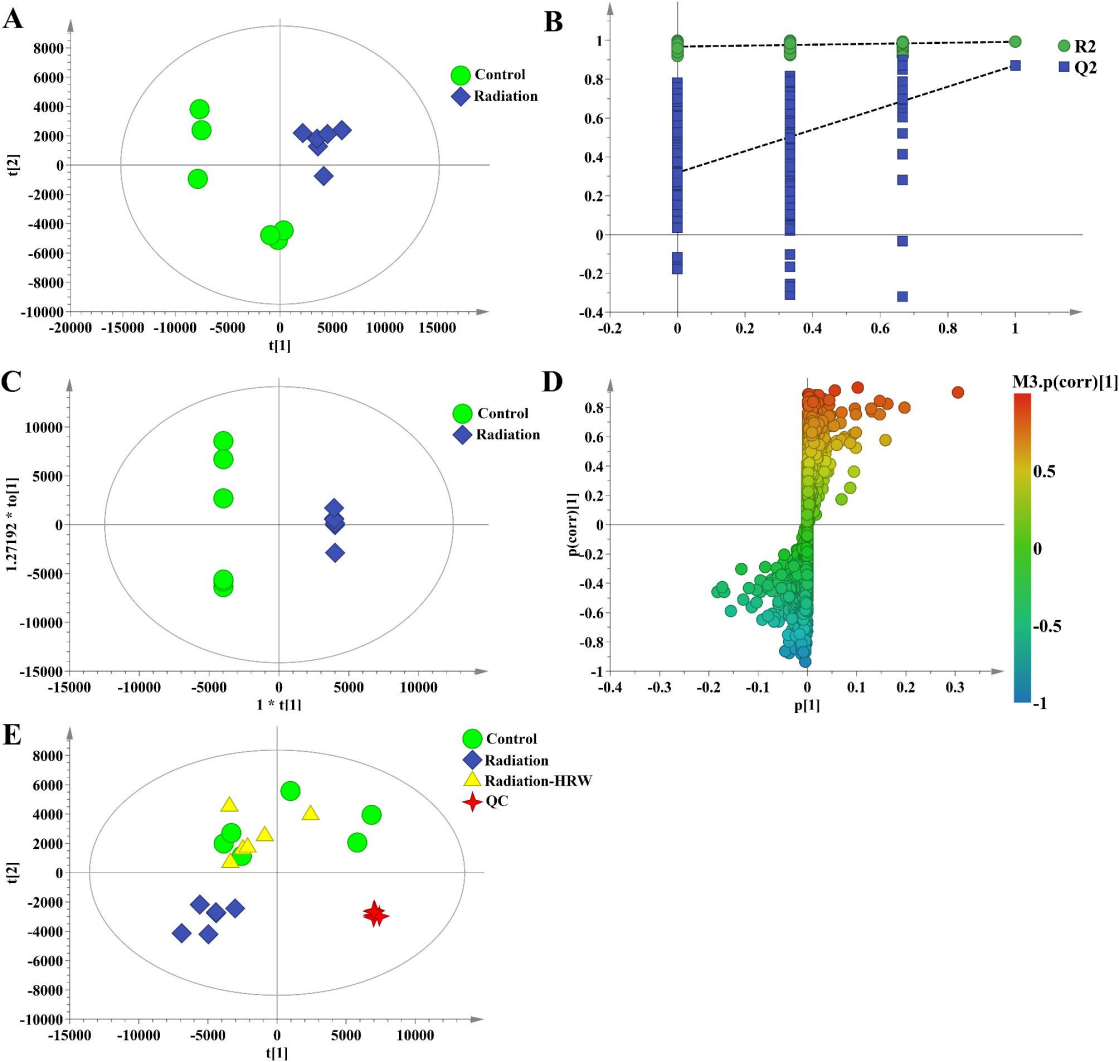
**Multivariate statistical analysis of LC-MS data**. (**A**) PLS-DA score chart of control and radiation group. (**B**) PLS-DA model verification diagram. (**C**) OPLS-DA score chart of control rats and model rats. (**D**) S-plot of control rats and model rats. (**E**) PLS-DA scores of brain tissue from different groups

PLS-DA was used for metabolic profiling to reduce the dimensionality of complex data obtained from brain tissue samples from the control and radiation groups. The model was clearly separated from the control group, indicating that this radiation had a significant effect on the endogenous metabolic spectrum of rat brain tissue (Fig. 2A). The PLS-DA model was then validated with an arrangement test (Fig. 2B). All the permutation values of R2 and Q2 were smaller than the original values, indicating that the established model was stable and reliable. We further analyzed the differences between groups using PLS-DA (Fig. 2E). The control group, the radiation group, and radiation-HRW group could be clearly distinguished, indicating that HRW was effective for radiation group rats. The QC samples were collected together in PLS-DA to demonstrate the robustness and applicability of the system and method.

Brain tissue sample data from control and model groups were analyzed with OPLS- DA to identify radiation-related differential metabolites. The combination of VIP > 1 (in S-plot) and *P* < 0.05 (in T-test) revealed a significant contribution of metabolites to homeostasis (Fig. 2 C and 2D). Compared with the control group, a total of 80 metabolites including LysoPE(18:1(11Z)/0:0), pantothenic acid, gamma-glutamylleucine, 1-phosphate ribose, stearoylethanolamide, etc., changed significantly in the model group. After the intervention of HRW, such changes were significantly adjusted (Table 1), indicating that HRW dramatically reversed the metabolic disorders of ionizing radiation rats.

**Table 1:** Statistics of differential metabolites recovered by HRW

No	Metabolites	m/z	Retention time (min)	Ion mode	Formula	Radiation vs. Control	Radiation-HRW vs. Radiation
1	Bisdiphosphoinositol tetrakisphosphate	704.8074441	0.74	Neg	C_6_H_20_O_24_P_8_	↓	↑
2	PC(20:1(11Z)/24:0)	880.7169796	0.74	Neg	C_52_H_102_NO_8_P	↓	↑
3	Choline	104.1073217	0.78	Pos	C_5_H_13_NO	↓	↑
4	alpha-methyl butyric Acid	120.1020555	0.78	Pos	C_5_H_10_O_2_	↓	↑
5	Ribothymidine	276.1187823	0.8	Pos	C_10_H_14_N_2_O_6_	↑	↓
6	2-N,6-N-Bis(2,3-dihydroxybenzoyl)-L-lysine	399.1192105	0.81	Neg	C_20_H_22_N_2_O_8_	↑	↓
7	Ribose 1-phosphate	229.0114577	0.82	Neg	C_5_H_11_O_8_P	↑	↓
8	Norophthalmic acid	274.1046339	0.82	Neg	C_10_H_17_N_3_O_6_	↑	↓
9	Caproic acid	134.1174357	0.84	Pos	C_6_H_12_O_2_	↓	↑
10	Drazoxolon	238.0377343	0.86	Pos	C_10_H_8_C_l_N_3_O_2_	↑	↓
11	S-Cysteinosuccinic acid	236.0230962	0.87	Neg	C_7_H_11_NO_6_S	↑	↓
12	Succinylproline	233.1129797	0.88	Pos	C_9_H_13_NO_5_	↓	↑
13	Glutathionate(1-)	308.090742	0.88	Pos	C_10_H_17_N_3_O_6_S	↑	↓
14	gamma-Glutamylglutamic acid	275.0886761	0.89	Neg	C_10_H_16_N_2_O_7_	↑	↓
15	Maleic hydrazide	113.0348169	0.89	Pos	C_4_H_4_N_2_O_2_	↓	↑
16	(4 S,5 S)-4,5-dihydroxy-2,6-dioxohexanoic acid	351.0571659	0.89	Neg	C_6_H_8_O_6_	↑	↓
17	Itaconic acid	175.0237955	0.89	Neg	C_5_H_6_O_4_	↑	↓
18	Aristolodione	306.0765161	0.89	Neg	C_18_H_13_NO_4_	↑	↓
19	Isobutyric acid	175.098179	0.91	Neg	C_4_H_8_O_2_	↑	↓
20	D-Lysine	191.1036811	0.94	Neg	C_6_H_14_N_2_O_2_	↓	↑
21	Propaphos	343.0535487	0.95	Pos	C_13_H_21_O_4_PS	↓	↑
22	ADP-ribose	604.0705895	1.05	Neg	C_15_H_23_N_5_O_14_P_2_	↑	↓
23	a-L-threo-4-Hex-4-enopyranuronosyl-D-galacturonic acid	351.0571152	1.17	Neg	C_12_H_16_O_12_	↑	↓
24	D-Glucuronic acid	175.0238612	1.17	Neg	C_6_H_10_O_7_	↑	↓
25	Glutathione	306.0763654	1.23	Neg	C_10_H_17_N_3_O_6_S	↑	↓
26	Letrozole	308.0905898	1.24	Pos	C_17_H_11_N_5_	↑	↓
27	3,4,5-trihydroxy-6-(2-oxo-1,3-diphenylpropoxy)oxane-2-carboxylic acid	385.1282845	1.24	Pos	C_21_H_22_O_8_	↑	↓
28	Pseudouridine	245.0766217	1.27	Pos	C_9_H_12_N_2_O_6_	↑	↓
29	(R)-3-hydroxybutyrylcarnitine	248.1490203	1.28	Pos	C_11_H_21_NO_5_	↓	↑
30	[2-hydroxy-5-(2-hydroxy-3-methoxy-3-oxopropyl)phenyl]oxidanesulfonic acid	310.0586886	1.38	Pos	C_10_H_12_O_8_S	↑	↓
31	2-(alpha-D-Galactosyl)-sn-glycerol 3-phosphate	143.0702116	1.39	Pos	C_7_H_12_O_4_	↓	↑
32	Cysteine-Homocysteine disulfide	299.037998	1.39	Neg	C_7_H_14_N_2_O_4_S_2_	↑	↓
33	Isoguanosine	284.0986875	1.42	Pos	C_10_H_13_N_5_O_5_	↓	
34	Guanosine	282.0844741	1.44	Neg	C_10_H_13_N_5_O_5_	↓	↑
35	L-Ascorbic acid-2-glucoside	383.0846362	1.44	Neg	C_12_H_18_O_11_	↑	↓
36	1,4-beta-D-Glucan	537.1685822	1.45	Pos	C_18_H_32_O_18_	↑	↓
37	Allopurinol-1-ribonucleoside	269.0878499	1.45	Pos	C_10_H_12_N_4_O_5_	↑	↓
38	Arabinosylhypoxanthine	267.0736398	1.45	Neg	C_10_H_12_N_4_O_5_	↑	↓
39	Allopurinol	137.0457685	1.45	Pos	C_5_H_4_N_4_O	↑	↓
40	5-fluoroindole-2-carboxylic Acid	357.0689298	1.45	Neg	C_9_H_6_FNO_2_	↑	↓
41	Elephantorrhizol	303.0502085	1.45	Neg	C_15_H_14_O_8_		↓
42	Saphenamycin	803.2361443	1.45	Neg	C_23_H_18_N_2_O_5_	↑	↓
43	Pyrocatechol glucuronide	571.1311958	1.46	Neg	C_12_H_14_O_8_	↑	↓
44	Isosorbide Dinitrate	281.0272222	1.49	Neg	C_6_H_8_N_2_O_8_	↑	↓
45	Carotamine	310.0588562	1.8	Pos	C_14_H_13_N_3_O_3_	↑	↓
46	Glutamyltyrosine	311.1235281	2.82	Pos	C_14_H_18_N_2_O_6_	↓	↑
47	Pantothenic Acid	220.1178617	3.09	Pos	C_9_H_17_NO_5_	↓	↑
48	S-(4,5-Dihydro-2-methyl-3-furanyl) ethanethioate	176.0739269	4.07	Pos	C_7_H_10_O_2_S	↑	↓
49	5β-cholanic Acid-3α, 12α-diol N-(2-sulphoethyl)-amide	538.2619276	4.20	Pos	C_26_H_45_NO_6_S	↑	↓
50	gamma-Glutamylleucine	261.1444168	4.55	Pos	C_11_H_20_N_2_O_5_	↓	↑
51	LysoPE(0:0/18:1(11Z))	480.3084895	11.46	Pos	C_23_H_46_NO_7_P	↓	↑
52	LysoPE(18:1(11Z)/0:0)	480.3083533	11.61	Pos	C_23_H_46_NO_7_P	↓	↑
53	Stearoylethanolamide	328.3207028	14.15	Pos	C_20_H_41_NO_2_	↓	↑
54	4-(beta-Acetylaminoethyl)imidazole	154.0973617	15.53	Pos	C_7_H_11_N_3_O	↑	↓

### Regulation of key metabolite-related metabolic pathways by HRW

**Fig. 3:**
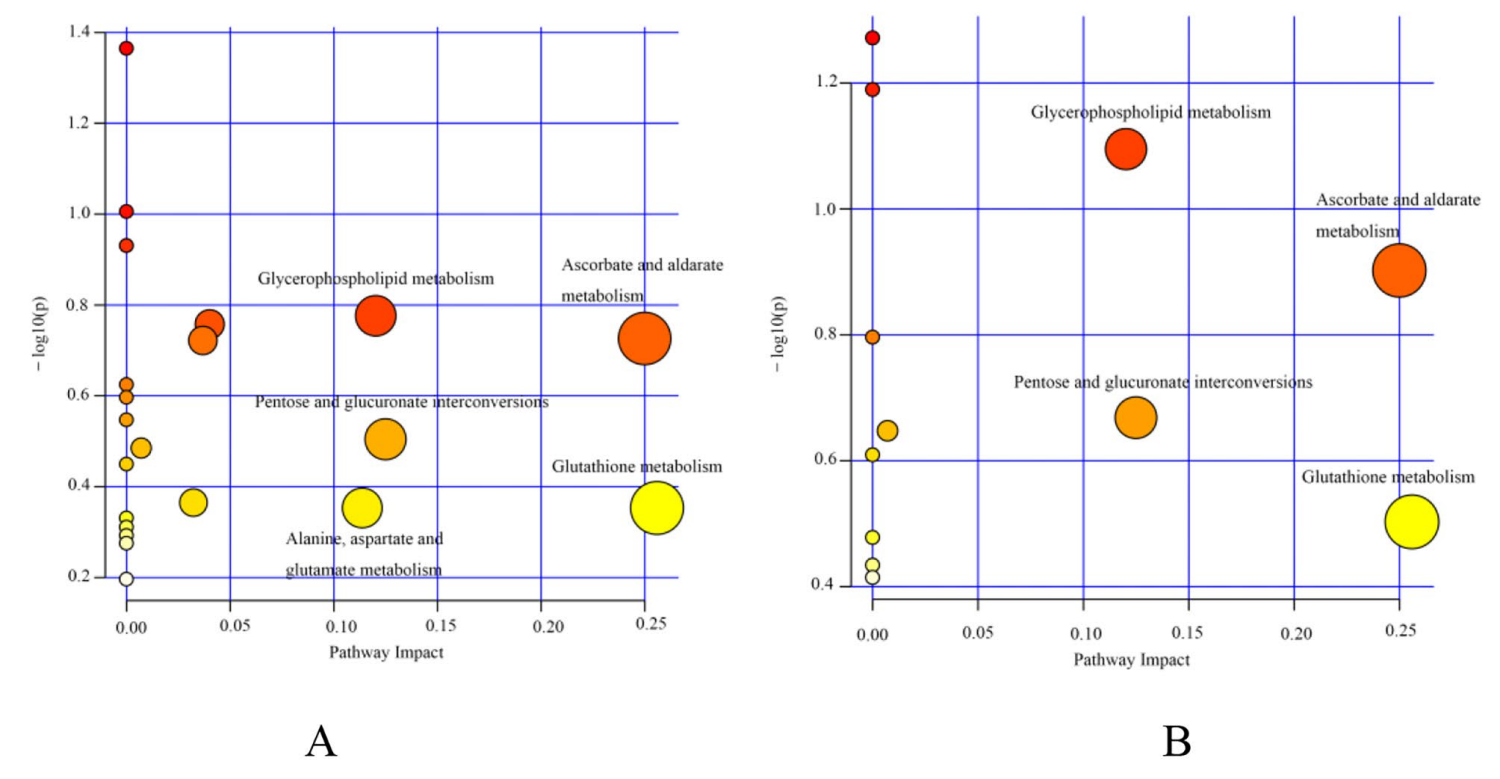
**Metabolic pathway analysis of MetPA**. (**A**) Analysis of ionizing radiation-related metabolic pathways. (**B**) Analysis of HRW-regulated metabolic pathways

To explore the metabolic pathways of ionizing radiation rats, differential metabolites were introduced into MetaboAnalyst for pathway enrichment analysis. Pathway effect > 0.1 was considered to be a key metabolic pathway (the results are shown in Fig. 3), and five metabolic pathways related to ionizing radiation were identified, including: glutathione metabolism, ascorbic acid and aldonic acid metabolism, pentose and glucuronic acid interconversion, glycerophospholipid metabolism, alanine, aspartic acid and glutamic acid metabolism. Four metabolic pathways (glutathione metabolism, ascorbic acid and aldonic acid metabolism, pentose and glucuronic acid interconversion, and glycerophospholipid metabolism) are specifically regulated by HRW. The above results suggest that HRW may improve ionizing radiation by regulating multiple metabolic pathways. It reflects the multi-objective and multi-pathway mechanism of HRW.

### Integrative analysis of metabolomics and network pharmacology

**Fig. 4:**
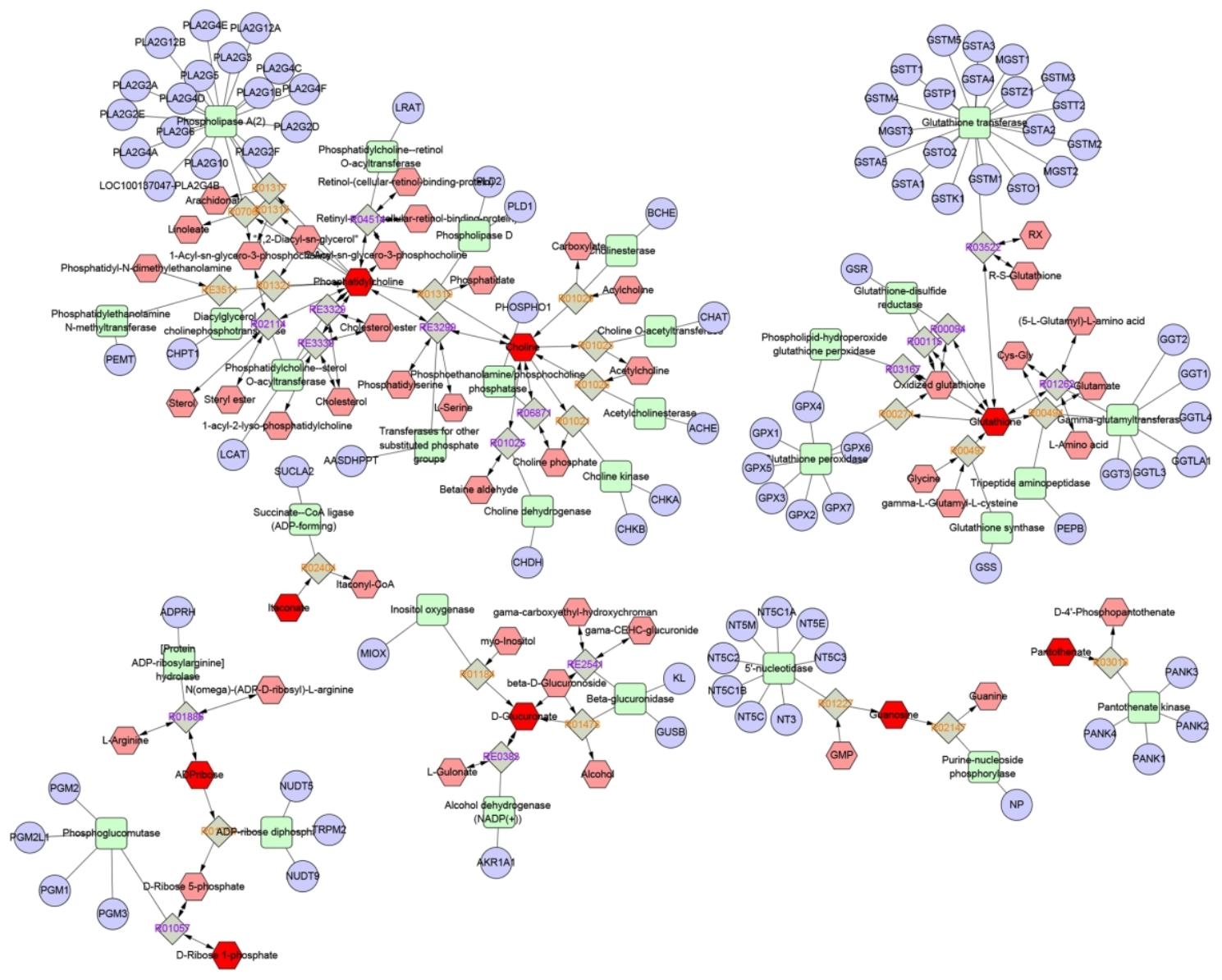
**The “active ingredient-target/gene-metabolite” biological network constructed by Cytoscape**. (Blue nodes: metabolite-related target genes, red nodes: differential metabolites.)

An interaction network based on metabolomics and network pharmacology was constructed to fully understand the mechanism of HRW against ionizing radiation (Fig. 4). Differential metabolites were introduced into the Cytoscape’s MetScape plug-in to collect a compound-response-enzyme-gene network. By matching potential targets identified in network pharmacology to genes from the MetScape analysis, we identified one key target. The key relevant metabolites are phosphatidylcholine, choline and glucuronide salts. The pathways affected are glutathione metabolism, ascorbic acid and aldehyde acid metabolism, pentose and glucuronic acid interconversion, and glycerophospholipid metabolism. They may play an important role in the therapeutic effects of FWR on ionizing radiation.

### Pathway analysis

**Fig. 5:**
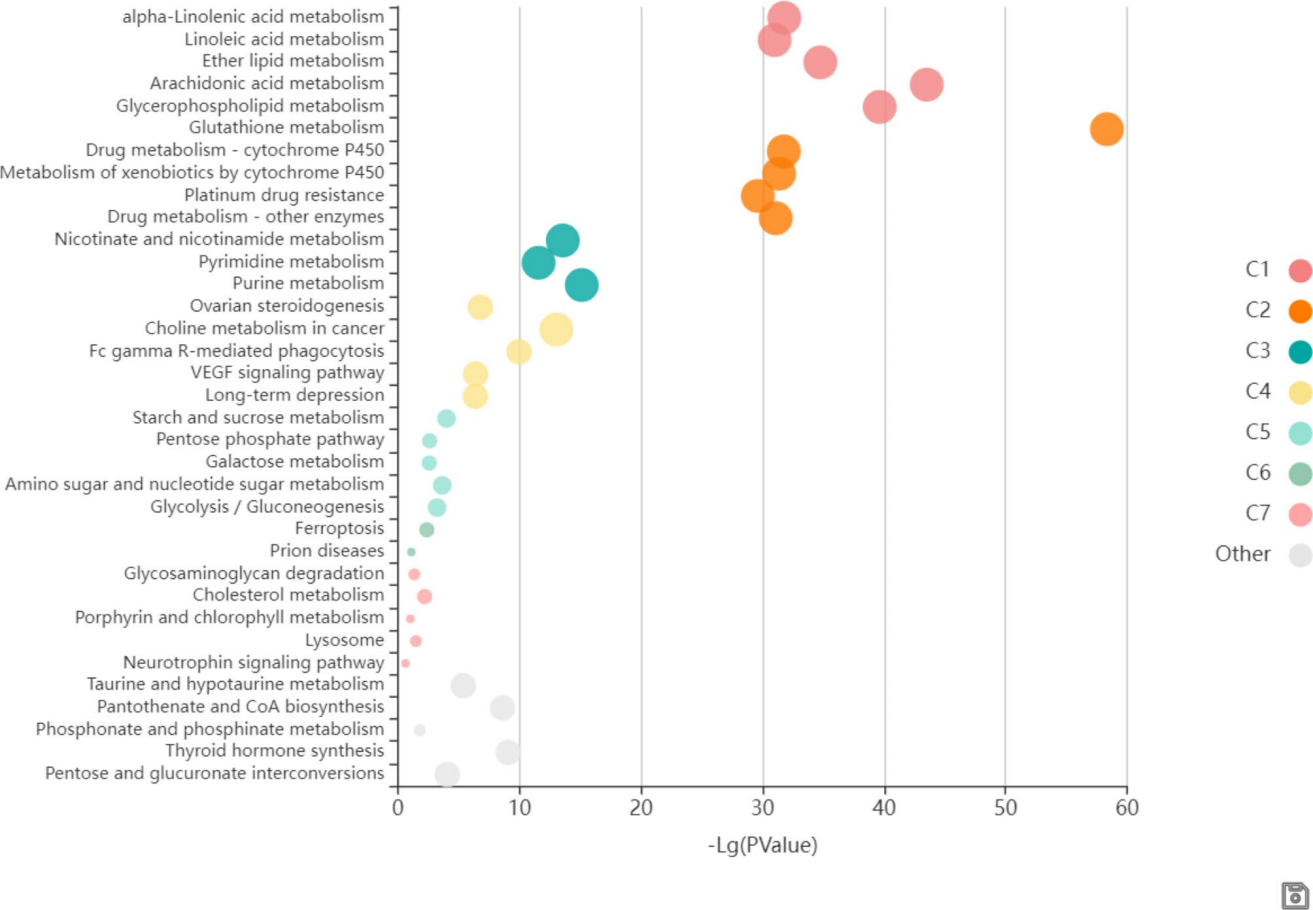
The KEGG enrichment analysis of HRW regulation-related targets

The KEGG enrichment analysis was performed on the targets and genes associated with recovery of differential metabolites, and the functions of these 93 key target genes were predicted and analyzed. The KEGG enrichment results showed that key signaling pathways sensitive to ionizing radiation, such as glutathione metabolism (C2), arachidonic acid metabolism(C1), glycerophospholipid metabolism(C1), and ether lipid metabolism(C1) were significantly enriched [18]. There were interactions between these signal pathways that can synergistically affect pathways that in turn, synergistically affected the HRW treatment with ionizing radiation (Fig. 5).

### Therapeutic performance evaluation of biomarkers

**Fig. 6:**
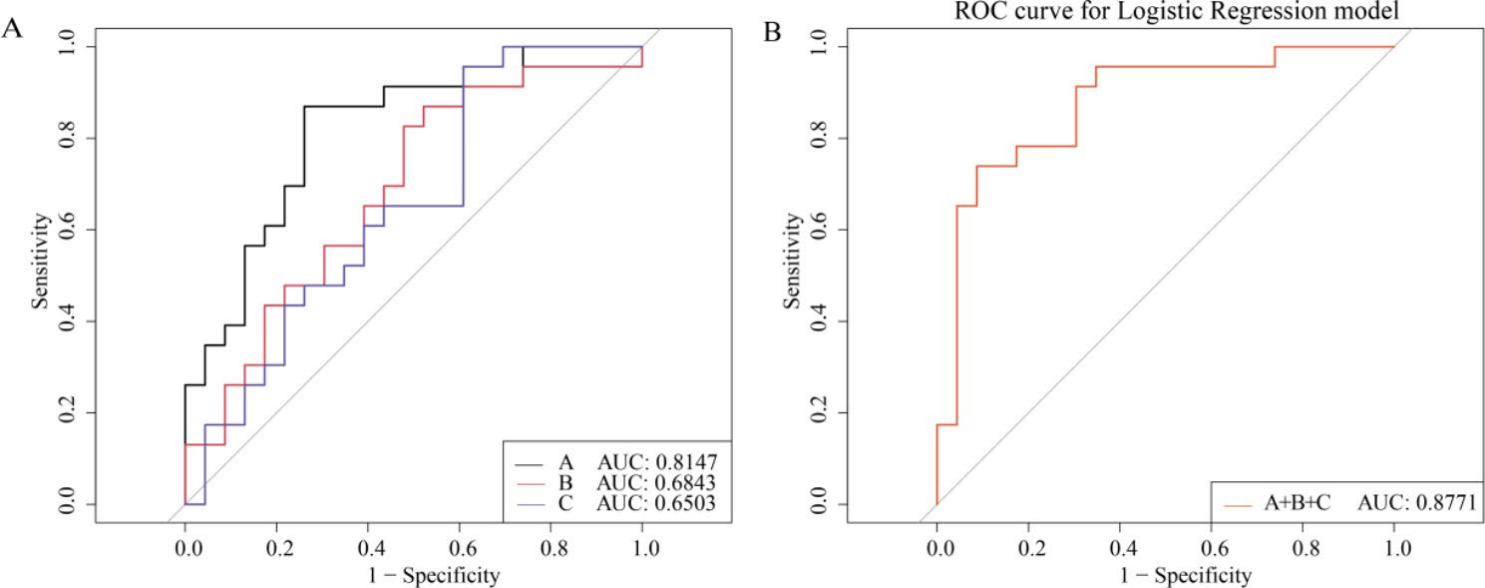
**ROC curves of the combined markers used to evaluate the effect of the HRW treatment**. (**A**) Phosphatidylcholine; (**B**) Choline; (**C**) D-Glucuronate. The combination of phosphatidylcholine, choline, and D-Glucuronate, and had the highest AUC = 0.8771

Brain tissue biomarkers are the cornerstone of ionizing radiation diagnosis, and their function was assessed by ROC analysis. A total of three metabolites were selected as potential biomarkers including phosphatidylcholine, choline, and D-glucuronate. Figure 6 A shows that the AUC for phosphatidylcholine, choline, and D- glucuronide were 0.8147, 0.6843, and 0.6503, respectively. In addition, using a biomarker combination that provided greater predictive power than a single biomarker, the combined result for phosphatidylcholine, choline, and D-glucuronide was 0.8771 by logistic regression analysis, as shown in Fig. 6B. These metabolites may be potential biomarkers of HRW in ionizing radiation therapy.

## Discussion

Radiation remains a health threat and safety hazards, and. radiation protection agents are attracting increasing attention. WR-2721 is the only FDA-approved radioprotective agent that can prevent radiation-induced damage, especially hydroxyl radical damage. WR-2721 can cause dizziness, vomiting and other adverse reactions. Therefore, there is an urgent need to develop an effective and safe radioprotectants. Hydrogen is a safe, convenient, non-toxic substance with little or no side effects and selective antioxidant function. Hydrogen scavenging prevents oxidative stress-induced damage [19, 20]. Hydrogen has a protective effect on various tissues from radiation damage. For example, intake of hydrogen-rich water after radiation can alleviate damages to the immune system, skin, hematopoietic system, small intestine, and heart by reducing reactive oxygen species (ROS) [21–25]. Their selective neutralization of harmful oxygen radicals has attracted increasing attention in the field of radiation protection. Hydrogen can cross the blood-brain barrier and quickly cross cell membranes to interact with toxic ROS. However, the material basis and potential mechanisms of hydrogen utilization in many neurological diseases remain unclear.

In our previous study, a single partial whole-brain irradiation with a 6-MeV electron beam of 30 Gy could successfully induce cognitive dysfunction in rats [26]. Compared with control rats, the number of new neurons decreased by 67% on the 7th day after 30 Gy whole brain irradiation [27]; and on the 30th day after irradiation, there were no surviving new neurons, indicating that irradiation with 30 Gy successfully induced acute cognitive dysfunction. In this study, a single 30 Gy electron beam whole brain irradiation was used to establish a model of cognitive dysfunction in rats. Then, we investigated the effects of HRW on cognitive function and its possible mechanism using Metabolomics combined with biological networks. The results showed that after HRW treatment, the cognitive dysfunction of rats was alleviated, and the protective effect was related to the regulation of phosphatidylcholine, choline, and D-Glucuronate. The results of brain metabolomics showed that HRW can significantly regulate the levels of 54 different metabolites, including glutathione metabolism, ascorbate and aldarate metabolism, pentose and glucuronate interconversions, and glycerophospholipid metabolism. These pathways were discussed below.

Pyroglutamic acid is a cyclized derivative of L- glutamic acid produced by non-enzymatic reaction of glutamic acid, glutamine and γ-glutamine peptide. It can also be generated by the reaction of γ-glutamyl cyclotransferase with amino acids. Acquired pyroglutamic acid deficiency (penicillins) and glutathione deficiency (e.g., malnutrition or sepsis) often result in moderate to severe encephalopathy clinically [28]. In our study, decreased levels of relevant metabolites were observed to decrease in ionizing radiation rats, and these metabolites could be restored by HRW, indicating that glutathione metallurgy may be the mechanism by which HRW treats ionizing radiation-induced brain injury.

Cognitive impairment seriously affects people’s physical and mental health and also causes a substantial economic burden [29]. This study showed that HRW can regulate the glycerophospholipid metabolism, which is the most abundant phospholipid in the body, and can activate the phospholipase A2 pathway to release arachidonic acid, thereby regulating the metabolism of glycerophospholipids and improve depression [30]. In addition, ascorbate and aldarate metabolism, pentose and gluconate interconversions are closely related to cognitive impairment [31, 32]. Our study found that HRW can improve ionizing radiation-induced brain cognitive impairment by modulating these pathways.

Although this study systematically explained the mechanism by which HRW improves cognitive impairment, there are limitations that require further study: This study only used non-targeted brain Metabolomics and biological target network at the level of metabolites and metabolic pathways. This model could be easily exploited in future works for the elucidation of pharmacological effects and mechanisms using a multi-omics approach based on integrated transcriptomics, proteomics and metabolomics studies.Further studies warrant adding corresponding pathway inhibitors at the cellular and animal levels for verification. In depth analysis though might be worth being conducted in future studies.

## Conclusions

In summary, the mechanisms of action of HRW on ionizing radiation were predicted through integrating biological networks and metabolomics. HRW may exert its potential effects by regulating 54 metabolites. In addition, glutathione metabolism, ascorbate and aldonic acid metabolism, pentose and glucuronate interconversions, and glycerophospholipid metabolism as well as the levels of phosphatidylcholine, choline, and D-Glucuronate are key metabolic pathways/metabolites for HRW function. These findings will help reveal the mechanism of HRW on ionizing radiation and provide a rational way to clarify this mechanism.

## Data Availability

All data generated or analyzed during this study are included in this published article.
